# A Decrease of Incidence Cases of Fumonisins in South Korean Feedstuff between 2011 and 2016

**DOI:** 10.3390/toxins9090286

**Published:** 2017-09-15

**Authors:** Juhee Park, Hansub Chang, Seungran Hong, Dongho Kim, Soohyun Chung, Chan Lee

**Affiliations:** 1 Advanced Food Safety Research Group, BrainKorea21 Plus, Department of Food Science and Technology, Chung-Ang University, 4726, Seodong-daero, Anseong-si 17546, Gyeonggi-do, Korea; bjhwngml@naver.com (J.P.); jjhs@korea.kr (H.C.); ppkpp112@naver.com (S.H.); 2 National Agricultural Products Quality Management Service, 141, Yongjeon-ro, Gimcheon-si 39660, Gyeongsangbuk-do, Korea; anoldmu@korea.kr; 3 Department of Integrated Biomedical and Life Science, Korea University, Seoul 02841, Korea; chungs59@korea.ac.kr

**Keywords:** mycotoxin, fumonisins, fumonisin B_1_, fumonisin B_2_, feedstuffs, compound feed, feed ingredients

## Abstract

Several plant pathogen *Fusarium* species produce fumonisins (FUMs); which can end up in food and feed and; when ingested; can exhibit harmful effects on humans and livestock. Mycotoxin intoxication by fumonisin B_1_ (FB_1_) and fumonisin B_2_ (FB_2_) can cause porcine pulmonary edema; leukoencephalomalacia in equines; esophageal cancer and birth defects by natural contamination. Herein; the occurrence of FB_1_ and FB_2_ in feedstuff (compound feed and feed ingredients) was investigated between 2011 and 2016 in South Korea. A total of 535 animal feed samples (425 compound feed samples and 110 feed ingredients) produced domestically were sampled four times between 2011 and 2016 (2011; 2012; 2014 and 2016) from feed factories in South Korea. The limit of detection (LOD) for FB_1_ and FB_2_ was 20 μg/kg and 25 μg/kg; respectively; and the limit of quantitation (LOQ) was 30 μg/kg and 35 μg/kg; respectively. The recovery range (%) was between 86.4% and 108.8%; and the relative standard deviation (RSD) (%) was 4.7–12.1%. Seven (swine feed samples) out of the 425 feed samples exceeded the European Union (EU) and South Korea commission regulations over the six-year test period; and no feed ingredients exceeded the guidelines.

## 1. Introduction

Toxins produced by filamentous fungi are called mycotoxins and can contaminate food and feed products [1]. Mycotoxin contamination can occur at various steps during food and feed production, including storage and distribution, and it decreases the quality of the product [2]. Indeed, mycotoxin contamination reduces the efficiency of livestock and crop production in many countries [3,4]. Furthermore, if a previously-contaminated ingredient is added to foodstuff or animal feed, human and animal health is threatened [5].

**Figure 1:**
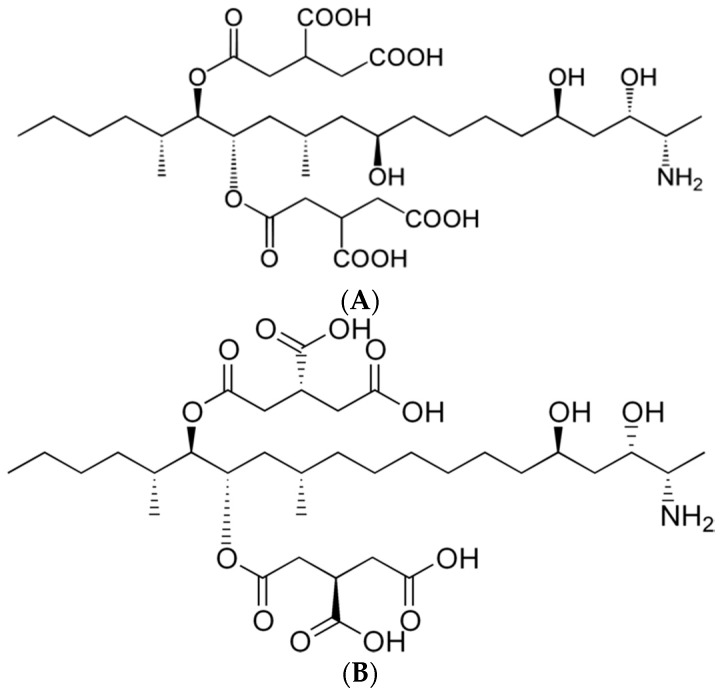
Chemical structures of fumonisin B_1_ (**A**) and B_2_ (**B**).

*Fusarium* species are toxigenic and pathogenic to plants or humans, and the trichothecenes and the fumonisins produced by them are the most well-known mycotoxins [6]. Fumonisins (FUMs) were first isolated from *F. moniliforme* in 1988 [7], and they can be produced by other *Fusarium* species such as *F. verticillioides*, *F. proliferatum* and *F. nygamai* [8]. FUMs are soluble in water, unlike other hydrophobic mycotoxins, and this property makes it difficult to study fumonisins [9]. Fumonisin B_1_ (FB_1_) is composed of two units of propane-1,2,3-tricarboxylic acid and a 2*S*-amino-12*S*,16*R*-dimethyl-3*S*,5*R*,10*R*,14*S*,15*R*-pentahydroxy-eicosane backbone connected by a diester bridge at the C-14 and C-15 hydroxy groups [10]. The chemical structures of FB_1_ and FB_2_ are shown in Figure 1.

These toxins can disrupt sphingolipid biosynthesis leading to serious problems in membrane biosynthesis because sphingolipids regulate cell growth and differentiation through participation in communication and interactions related to the cell [11,12]. They also cause leukoencephalomalacia, pulmonary edema, hydrothorax, apoptosis, hepatotoxic and carcinogenic effects in animals [7,13,14,15,16,17,18], as well as esophageal cancer in humans [19]. FB_1_ was assigned to group 2B, “possibly carcinogenic to humans”, by the International Agency for Research on Cancer [20].

Because of the serious damage that mycotoxins can cause to human and animal health, mycotoxins are an important concern for the feed industry, feed supply chain and international trade [21]. Binder et al. [22] reported that the amount and type of fungal contamination exhibits significant regional differences. For example, in their study, European samples were mostly contaminated by deoxynivalenol (DON), T-2 toxin and zearalenone (ZEN), but Asian and Pacific samples had aflatoxins (AFs), DON, FUMs and ZEN as major contaminants. In 2015, the levels of FB_1_ and glycated FB_1_ in a total of 30 compound feed samples were measured, and all measured concentrations of FB_1_ were below the regulation limits set by the European Union and the U.S. Food and Drug Administration [23].

Grains, grain byproducts and vegetable proteins found in animal feed are good nutrients for the fungi that produce mycotoxins. Therefore, mycotoxins in animal feed need to be closely monitored. In 2012, 18,640 tons of compound feed were distributed in Korea, and 15,350 tons of feed ingredients were purchased by Korea from many places including China, the USA, Europe, Canada, South Africa, South East Asia, Australia and India. Feed ingredients from different countries make it difficult to control mycotoxin levels in compound feed. In one study in Slovakia, a total of 50 poultry feed samples was collected during 2003 and 2004, and the level of FUMs was measured. FB_1_ and FB_2_ were detected in 49 samples (98.0%) and 42 samples (84.0%), respectively [24]. In another study, brewers’ grain, used as dairy cattle feed, was analyzed using high performance liquid chromatography (HPLC) in Brazil, and FUMs contaminated 72.5% (58) of the samples with rates between 50.3 and 908.5 μg/kg [25]. In China, six mycotoxins were quantified in 83 feed samples including FUMs, and 82.7% were contaminated [26].

According to the European Commission (EC), maximum levels for *Fusarium* toxins were established in products intended for animal feeding in 2006 [27], and the Food and Drug Administration (FDA) in the U.S. set guidance levels for FUMs in human food and animal feed in 2001 [28]. In South Korea, the guidance value for *Fusarium* mycotoxins in animal feed was established based on guidelines of the EC and the results of studies in Korea. The guidelines for the EC and South Korea are summarized in Table 1.

The object of this study was to confirm the contamination level of FUMs in feed and feed ingredients and to compare the level of FUMs with the guidelines for animal feed in South Korea. For this purpose, we collected feed samples over the course of six years, analyzed the levels of FUMs contamination and assessed the risk of FUMs contamination in animal feed in Korea.

## 2. Results

### 2.1. Method Validation

**Figure 2:**
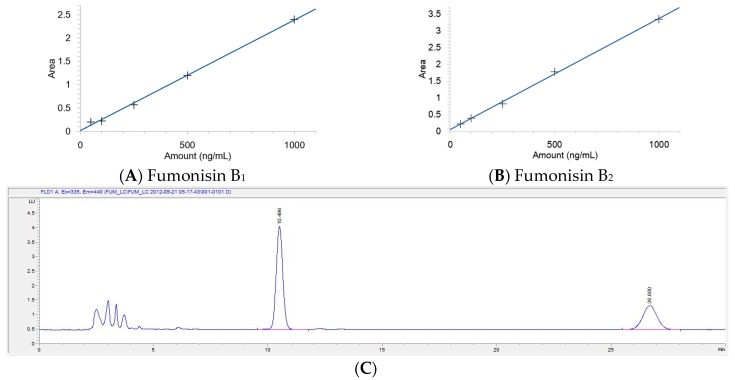
Calibration curve for fumonisin B_1_ (**A**) and B_2_ (**B**) and the HPLC chromatogram (**C**).

**Figure 3:**
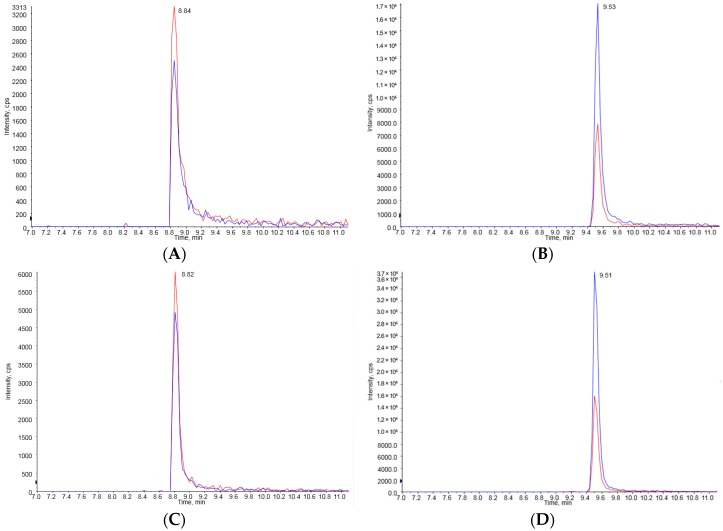

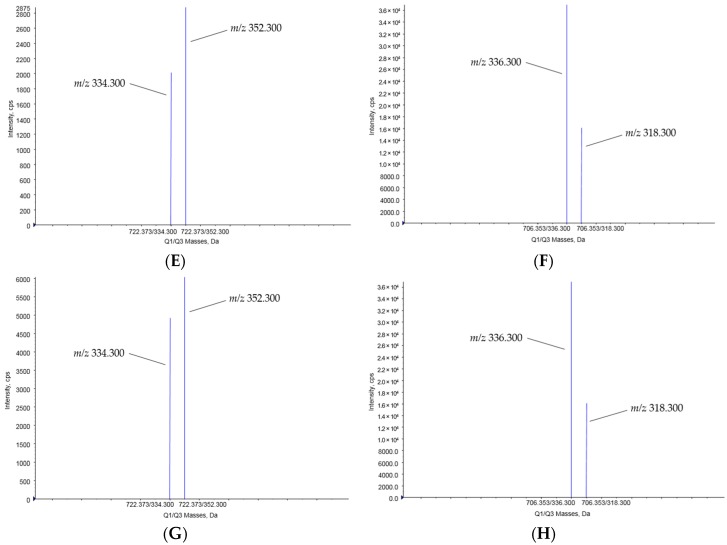
Identification of FUMs by LC-MS/MS. An extracted ion chromatogram of standard FB_1_ and FB_2_ at 250 ppb (**A**,**B**) and FUMs in a feed sample (**C**,**D**). The ion spectrum (product ion) of standard FB_1_ and FB_2_ (**E**,**F**) and those in a feed sample (**G**,**H**).

Fumonisin B_1_ and B_2_ could be clearly separated from any potential interference in the HPLC chromatogram after pretreatment with an immunoaffinity column (IAC). The LOD and LOQ were estimated at 20 μg/kg and 30 μg/kg for FB_1_ and 25 μg/kg and 35 μg/kg for FB_2_, respectively. The regression coefficient was over 0.999 in the solvent-based standard curve for FUMs (Figure 2A,B). The HPLC chromatogram of the standard solutions of FUMs spiked into an animal feed sample did not show any interfering peaks in the HPLC chromatogram (Figure 2C). Therefore, this analytical method was suitable for the analysis of FUMs. The accuracy for FUMs was expressed as the average recovery ratio in the recovery test. Precision was calculated from the same tests and expressed as percent relative standard deviation (%RSD). The range of recovery ratio was 86.4–108.8%, and %RSD was 4.7–12.1% (Table 2). These results satisfied the commission regulation for FUMs in the EU that defines an accuracy of 70–120% and %RSD for precision under 20% [30]. The results of the method validation study are summarized in Table 2. After analysis of FUMs in the HPLC chromatogram, LC-MS analysis was further applied to identify FUMs with its extracted ion chromatogram (XIC) and spectrum of mass (Figure 3). Extracted ion chromatograms of FB_1_ and FB_2_ in a standard solution (Figure 3A,B) and in a representative sample (Figure 3C,D) exhibited the same results as the precursor ion (*m*/*z* 722.373 and 706.353 [M + H]^+^ for FB_1_ and FB_2_, respectively); two product ions (*m*/*z* 334.3 and 352.3 for FB_1_ and *m*/*z* 336.3 and 318.3 for FB_2_) from the precursor ion (*m*/*z* 722.373 and 706.353 [M + H]^+^ for FB_1_ and FB_2_, respectively) of FB_1_ and FB_2_ (Figure 3E,F) matched exactly with those from the sample (Figure 3G,H).

### 2.2. Occurrence of FUMs in Compound Feed Samples between 2011 and 2016

The contamination levels of FUMs were estimated from 425 compound feed samples consisting of cattle feed (165), pig feed (140) and poultry feed (140), which were collected in 2011, 2012, 2014 and 2016. FB_1_ was detected in 90.6% of all of the compound feed samples; it was detected in 97.9% of cattle feed samples, followed by 90.7% of poultry feed samples and 82.9% of pig feed samples. FB_2_ also contaminated 63.3% of all compound feed samples: 78.6% of cattle feed samples, 45.7% of pig feed samples and 65.0% of poultry feed samples. Detailed data including mean contamination level of FUMs by feed type are shown in Table 3. Almost all compound feed samples were contaminated with FB_1_, and the mean contamination level in all of the compound feed samples was 1448.1 μg/kg, with the level of FB_1_ in the range of 36.4–14,876.8 μg/kg. FB_2_ was detected in over half of compound feed samples. Its mean contamination level was 210.8 μg/kg with the contamination level in the range of 14.9–9235.8 μg/kg.

The mean concentration level of FB_1_ in cattle feed, pig feed and poultry feed was 1779.0, 1171.1 and 1382.5 μg/kg, respectively, so cattle feed had the highest mean contamination level among the three types of compound feed. In the case of FB_2_, the highest mean contamination level was shown in poultry compound feed. There was no statistically significant difference between the three groups of compound feed samples in a one-way analysis of variance (ANOVA, *p* < 0.05) (Table 4).

**Figure 4:**
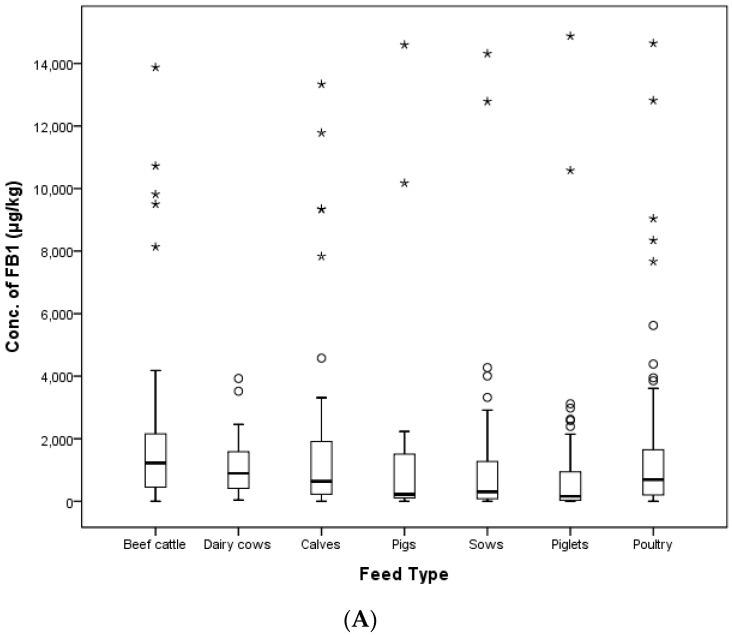

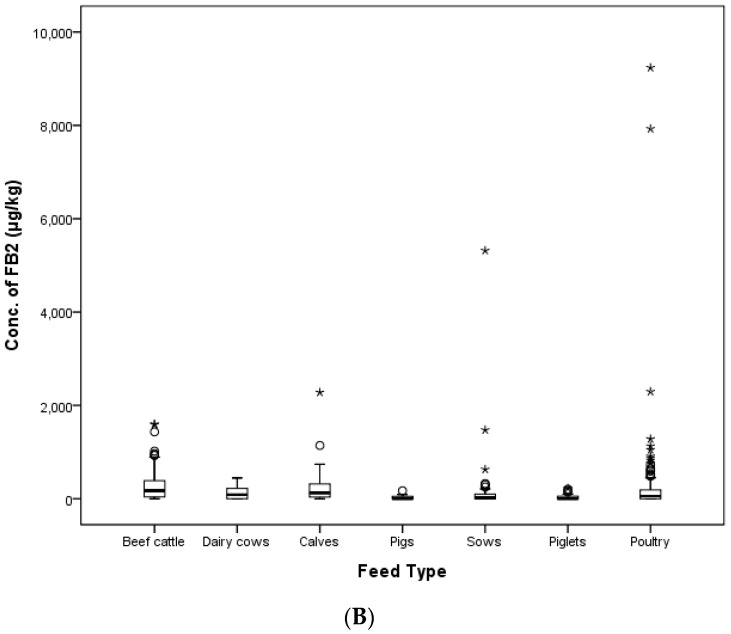
Distribution of FB_1_ (**A**) and FB_2_ (**B**) in compound feed types (box-plot: whiskers at minimum and maximum, box at P25 and P75 with the line at P50; ° values above the 75th percentile plus 1.5-times the inter-quartile distance; * values above the 75th percentile plus 3.0-times the inter-quartile distance).

The highest contamination value for FB_1_ (14,876.8 μg/kg) was detected in a weanling piglet feed, and this sample had an FB_2_ level of 221.1 μg/kg. The sum of these value was over the EC and Korean regulation limits (5000 μg/kg). In addition, two growing pig feed samples, two gestating sow feed samples, a lactating sow feed sample, a sucking piglet feed sample and a weanling piglet feed sample exceeded the EC and Korean limits. Among all of the animal compound feed, only several pig feeds (1.41%) were above the limit. In the case of FB_2_, the highest level of FB_1_ (8343.4 μg/kg) was detected in an early laying hens’ feed, which shows FB_2_ co-contamination (1048.2 μg/kg), and the highest level of FB_2_ (9235.8 μg/kg) was detected in an early broiler feed exhibiting 446.1 μg/kg of FB_1_ content. The distribution of FUMs according to the type of compound feed is shown in Figure 4.

**Figure 5:**
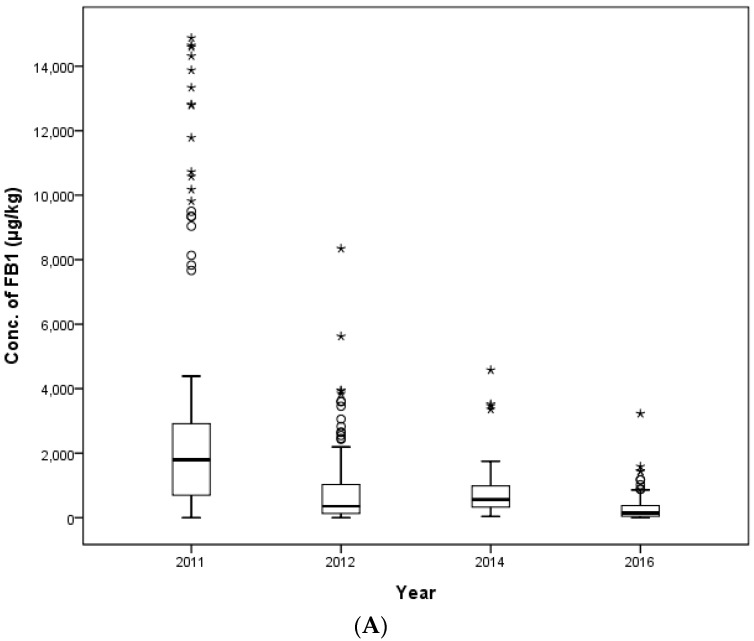

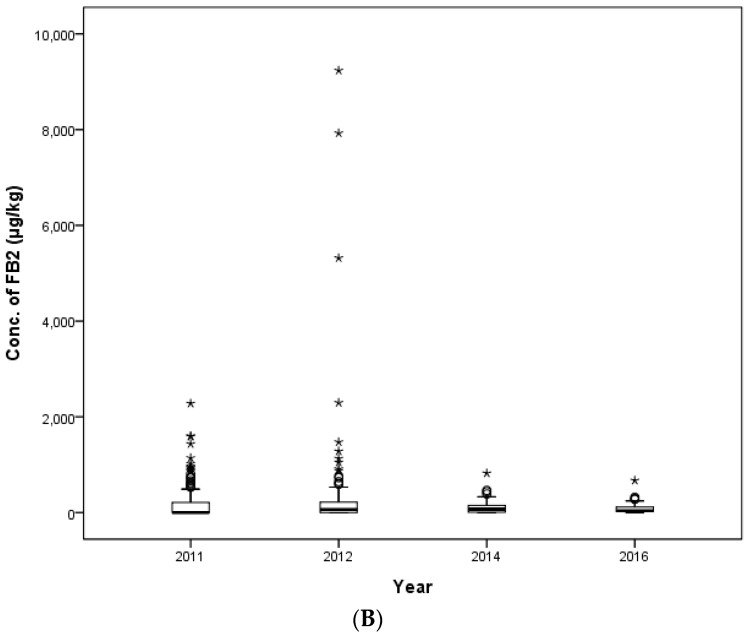
Distribution of FB_1_ (**A**) and FB_2_ (**B**) in compound feed between 2011 and 2016 (box-plot: whiskers at minimum and maximum, box at P25 and P75 with the line at P50; ° values above the 75th percentile plus 1.5-times the inter-quartile distance; * values above the 75th percentile plus 3.0-times the inter-quartile distance).

The distributions of FUMs by year of sampling are shown in Figure 5. In 2011, 2012, 2014 and 2016, the mean contamination level of FB_1_ was 2817.5, 805.1, 839.2 and 341.2 μg/kg, respectively, and for FB_2_, it was 192.5, 324.4, 112.1 and 84.1 μg/kg, respectively. In 2011, a weanling piglet feed sample showed the highest level of FB_1_ (14,876.8 μg/kg), and a middle beef calf sample showed the highest level of FB_2_ (2277.9 μg/kg). In 2012, the highest level of FB_1_ (8343.4 μg/kg) was detected in an early laying hens’ feed, and the highest level of FB_2_ (9235.8 μg/kg) was detected in an early broiler feed. In 2014, a middle beef calf sample had the highest level of FB_1_ (4576.1 μg/kg), and an early broiler feed showed the highest level of FB_2_ (821.2 μg/kg). Lastly, in 2016, the highest contamination level was evaluated in a middle dairy calf feed with an FB_1_ concentration of 3225.0 μg/kg and an FB_2_ concentration of 670.0 μg/kg.

### 2.3. Occurrence of FUMs in Feed Ingredients between 2011 and 2016

One-hundred and ten feed ingredient samples were collected four times over six years: in 2011, 2012, 2014 and 2016. The contamination levels of these samples were evaluated using HPLC. The collected feed ingredient samples were composed of grains (seven samples), grain by-products (bran, 27 samples), meal (vegetable protein, 55 samples), fibrous feed (nine samples), food by-products (eight samples) and other feed ingredients (one sample of beans, two samples of seeds/nuts and a mixed formulation sample). The detailed data related to the collected feed ingredient samples are shown in Table 5.

The contamination rates of FB_1_ were 14.3% in grains, 70.4% in grain by-products (bran), 63.6% in meal, 33.3% in fibrous feed, 37.5% in food by-products and one bean sample. Overall, 56.4% of the tested feed ingredients were contaminated by FB_1_. In the case of FB_2_, the contamination rates were 48.1, 45.5, 44.4 and 12.5 in grain by-products (brans), meal, fibrous feed and food by-products, respectively. However, grains, seeds/nuts and mixed formulations were not contaminated by FB_2_, so 40.0% of the total samples exhibited contamination with FB_2_. The mean contamination levels of FUMs were 624.9 and 169.3 μg/kg for FB_1_ and FB_2_, respectively. The FUMs concentrations in total feed ingredients ranged from 15.5–6357.5 μg/kg for FB_1_ and from 21.5–1798.9 μg/kg for FB_2_.

**Figure 6:**
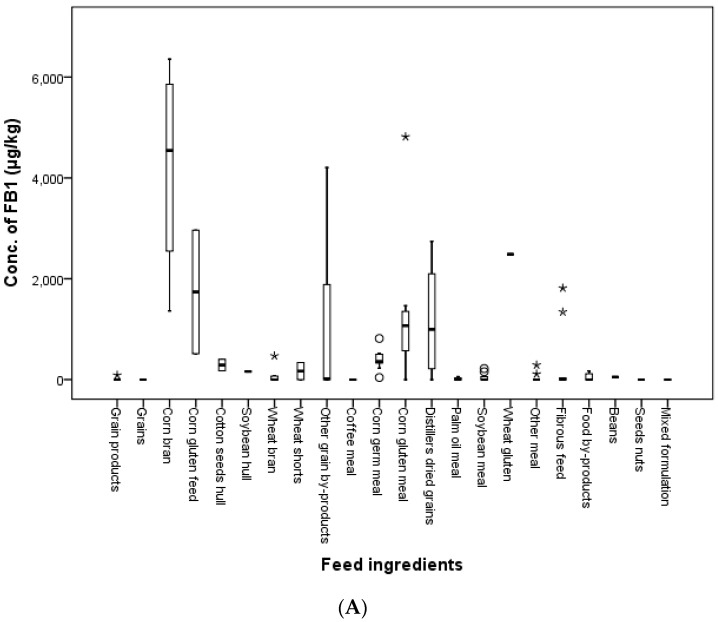

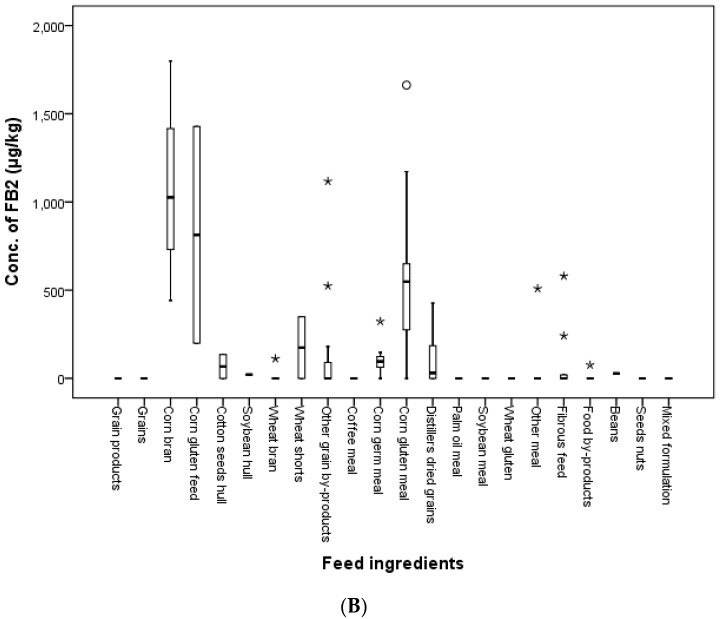
The distribution of FB_1_ (**A**) and FB_2_ (**B**) in feed ingredients (box-plot: whiskers at minimum and maximum, box at P25 and P75 with the line at P50; ° values above the 75th percentile plus 1.5-times the inter-quartile distance; * values above the 75th percentile plus 3.0-times the inter-quartile distance; - values mean median value for each feed ingredient).

The calculated mean contamination values of FB_1_ were 12.5, 1224.8, 580.8, 354.0 and 50.5 μg/kg in grains, grain by-products, meal, fibrous feed and food by-products, respectively; and the mean contamination values of FB_2_ were 309.6, 169.4, 94.7 and 9.4 μg/kg in grain by-products, meal, fibrous feed and food by-products, respectively. The maximum contaminated concentrations of FB_1_ and FB_2_ were 6357.5 and 1798.9 μg/kg in corn bran, 4816.0 and 1662.7 μg/kg in corn gluten meal, 1815.0 and 580.0 μg/kg in fibrous feed and 169.5 and 75.5 μg/kg in food by-products, respectively; and only one grain product was contaminated by FB_1_ at 87.3 μg/kg. FUMs were not found in seeds/nuts or mixed formulations. Forty-four percent of all feed ingredient samples had lower FB_1_ concentrations than the LOD, and 60.0% of the total feed ingredients had lower FB_2_ values than the LOD. The distributions of FB_1_ and FB_2_ in accordance with feed ingredients and class of feed ingredients are shown in Figure 6.

According to guidelines set by the EC and Korea, the limit for FUMs was 60,000 μg/kg in maize by-products. The maximum concentration of FUMs was observed in a corn bran sample with 8156.4 μg/kg FB_1_ + FB_2_. This value is under the limit in the EU and Korea. In other feed ingredient types, the maximum contamination values for FUMs were 6478.7 μg/kg in corn gluten meal and 1138.7 μg/kg in corn germ meal. Therefore, no feed ingredient samples exceeded the guidance level.

**Figure 7:**
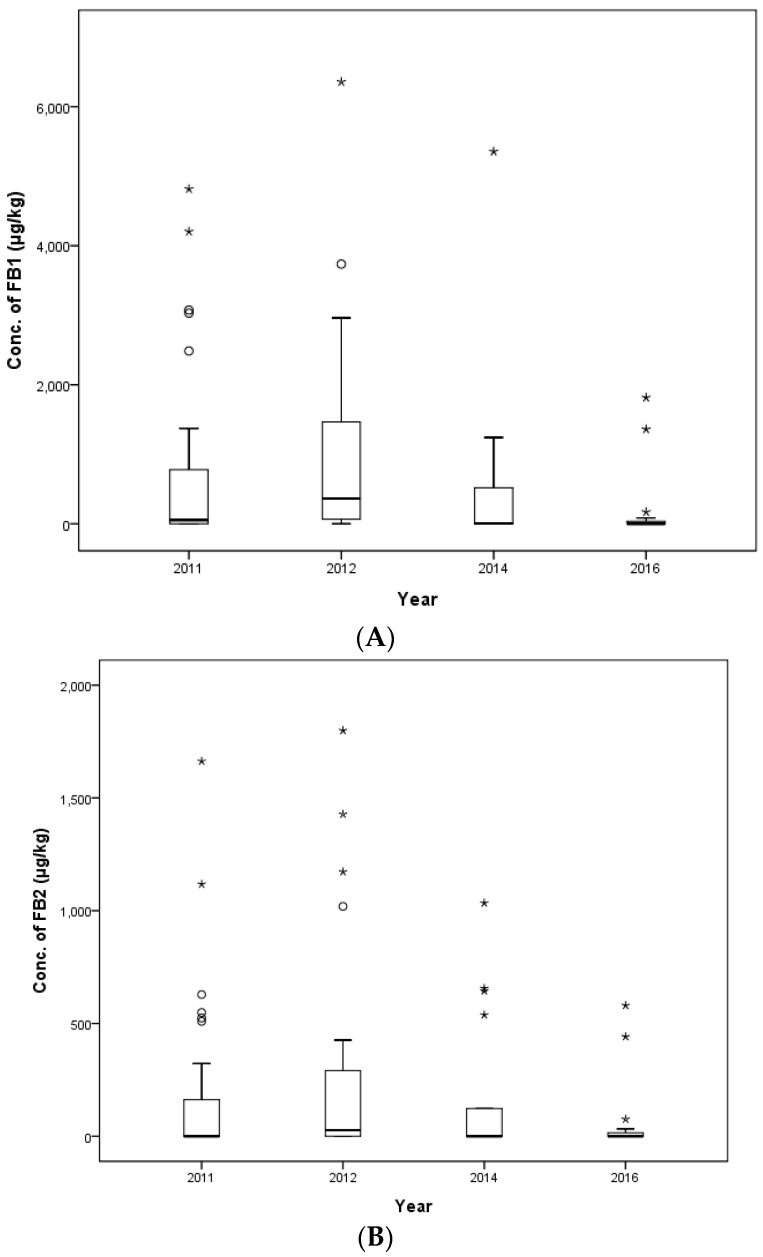
Distribution of FB_1_ (**A**) and FB_2_ (**B**) in feed ingredients across the years (box-plot: whiskers at minimum and maximum, box at P25 and P75 with the line at P50; ° values above the 75th percentile plus 1.5-times the inter-quartile distance; * values above the 75th percentile plus 3.0-times the inter-quartile distance).

The number of collected feed ingredient samples was 110 samples including 40 samples in 2011, 30 samples in 2012, 17 samples in 2014 and 23 samples in 2016. Mean contamination values were 680.6, 947.6, 559.2 and 155.1 μg/kg for FB_1_ and 161.0, 266.2, 177.4 and 51.7 μg/kg for FB_2_ in 2011, 2012, 2014 and 2016, respectively (Table 6). Maximum levels of FUMs were estimated as follows: 4816.0 μg/kg of FB_1_ and 1662.7 μg/kg of FB_2_ in corn gluten meal in 2011, 6357.5 μg/kg of FB_1_ and 1798.9 μg/kg of FB_2_ in corn bran in 2012, 5354.9 μg/kg of FB_1_ and 1033.9 μg/kg of FB_2_ in corn bran in 2014 and 1815.0 μg/kg of FB_1_ and 580.0 μg/kg of FB_2_ in fibrous feed in 2016. The occurrence of FUMs in each year (2011, 2012, 2014 and 2016) is shown in Figure 7. There was no statistically-significant difference year-to-year for feed ingredients in a one-way ANOVA (*p* > 0.05, Table 6).

## 3. Discussion

Many studies related to FUMs have been performed worldwide in order to determine contamination levels in compound feed. For example, Marquardt and Madhyastha [32] surveyed levels of various toxins in feed material and feedstuff worldwide. The highest incidence, with more than 50% positive samples, was found in North, South and Southeast Asia, South America, Southern Europe and Africa. A study in Kuwait [33] reported that all samples of poultry feed that they tested (53 samples) were contaminated with FUMs in the range of 220–6000 μg/kg with the mean contamination level of 2733 μg/kg. Similar results were reported by Greco et al. [34]: FUMs were detected in all of the 46 poultry feed samples with the concentration between 222 and 6000 μg/kg (median 1750 μg/kg). Kocasari et al. (2013) [35] analyzed 180 compound feed samples in Turkey for the contamination of FUMs; approximately 10.6% of samples were contaminated by FUMs, and the mean contamination value was measured at 3190 μg/kg with range of 2690–4960 μg/kg. In the Republic of South Africa, a total of 92 commercial compound feed samples was examined for contamination with various mycotoxins, and FUMs were detected in most of the compound feed samples [36]. Specifically, cattle feed was severely contaminated by FB_1_ and FB_2_. The mean contamination level of FUMs was 916 μg/kg, and the highest contamination level was 2999 μg/kg. In addition, the mean contamination value of FUMs in cattle, chicken and swine feed was 903, 975 and 313 μg/kg, respectively. The range of the contamination level of FB_2_ was 103–673 μg/kg, which is lower than the contamination values for FB_1_. This research showed that no compound feed samples exceeded the guidance level of the EC. Another study [37] was conducted to determine the level of FUMs in a total of 30 pig feed samples. The mean contamination level of FUMs was 405 μg/kg, and 80% of the feed samples were contaminated by FUMs, ranging from 30–1043 μg/kg. The level of FUMs in all of samples was below 5000 μg/kg, the EC guidance value. Zachariasova et al. [38] conducted a study in order to quantify 56 mycotoxins in 343 samples of animal feed. Those samples were non-fermented and fermented feedstuff, feedstuff supplements and complex compound feed collected from the Czech Republic (256 samples) and the United Kingdom (87 samples) between 2008 and 2012. Ninety-five compound feed samples (for pigs, chickens and laying hens, birds, rodents and dairy cows) were analyzed among the collected 343 samples, and only 13 compound feed samples for dairy cows were contaminated with FB_1_; three compound feed samples for dairy cows were contaminated by FB_2_. The median level of FB_1_ was 10 μg/kg, and the maximum level was 58 μg/kg. In the case of FB_2_, the median was 9 μg/kg, and the maximum level was 14 μg/kg. Those results were much lower than the EC legislation. In that article, FUMs were detected below 100 μg/kg in all 278 samples. In Poland, a study [39] was performed to evaluate the level of several mycotoxins in 1384 samples including 480 complete feed samples during a four-year period. In that study, FUMs were present in 85.7% of the 14 complete feed samples analyzed. The maximum level of FUMs was measured at 1063 μg/kg. A total of 74 ready-to-consume feed samples for dairy cattle was collected in Konya, Turkey and the surrounding provinces [40]. Seven mycotoxins were analyzed in collected feed samples, and FUMs were the most common mycotoxin; 93.2% of samples had detectable levels of FUMs. The maximum detected level was 1208 μg/kg, which was below the 50,000 μg/kg maximum residue limit in Turkey. A similar study [41] was also performed in Turkey in which 22 cattle feed samples were collected every quarter over one year (total 88 samples) from some provinces in Turkey; 94.3% of those samples were contaminated by FUMs. The contamination level was between 0 and 3900 μg/kg. Samples collected in the summer exhibited a higher mean contamination level than in any other season.

In this study, the contamination level of FUMs was estimated in 425 feed samples consisting of 145 samples of cattle feed, 140 samples of pig feed and 140 samples of poultry feed collected in 2011, 2012, 2014 and 2016 in the Republic of Korea. FB_1_ and FB_2_ were detected in 90.6% and 63.3% of compound feed samples. FUMs mainly contaminated cattle feed (97.9% for FB_1_ and 78.6% for FB_2_), followed by poultry feed (90.7% for FB_1_ and 65.0% for FB_2_) and pig feed (82.9% for FB_1_ and 45.7% for FB_2_). The incidence of FB_1_ contamination in pig feed was similar to the incidence of FUMs in pig feed reported by Pleadin et al. (2012) [37] (80%). The mean concentration of FB_1_ was 1779.0 μg/kg for cattle feed, 1171.1 μg/kg for swine feed and 1382.5 μg/kg for poultry feed, and the mean concentrations of FB_2_ were 251.8, 90.5 and 287.9 μg/kg for cattle, swine and poultry feed, respectively. The mean concentrations of FB_1_ and FB_2_ were 1382.5 and 287.9 μg/kg in poultry feed, which were lower than the estimated value (2733 μg/kg) in poultry feed in Kuwait [33]. The maximum contamination value of FUMs was detected at 15,098 μg/kg in a weanling piglet feed sample. This contamination level was over the EC and Korean regulation limit (5000 μg/kg). The concentration of FUMs in six other pig feed samples, as well as the weanling piglet feed mentioned above also exceeded the EC and Korean guidance level. In this study, the concentration ranges of FB_1_ (36.4–14,876.8 μg/kg) and FB_2_ (14.9–9235.8 μg/kg) contamination were broader than the ranges from Kuwait (220–6000 μg/kg) [33] and Turkey (2690–4960 μg/kg) [35].

In Kuwait [33], wheat bran, soybean meal and corn feed ingredients were contaminated by FUMs (89.8% of samples), and the mean contamination value was 2040 μg/kg with a range of 0–6000 μg/kg. Another study [42] was conducted to determine the occurrence of various mycotoxins including fumonisins. A total of 7049 samples consisting of corn, soybean/soybean meal, wheat, dried distillers’ grains with solubles (DDGS) and finished feed samples was collected from the Americas, Europe and the Asia-Pacific region. FUMs contaminated most of these samples with mean contamination values of 1965 μg/kg. Corn from South America and Southern Europe was mainly contaminated with FUMs, and the contamination incidence was 92% and 90%, respectively. The mean contamination levels in both regions differed; the mean level of FUMs in South American samples (3226 μg/kg) was higher than that in Southern European ones (2271 μg/kg). In 2011–2014, a total of 1384 samples consisting of 295 maize samples, 143 maize silage samples, 466 small grain cereal samples and 480 complete feed samples was collected in Poland [39]. Those samples were used to estimate the level of various mycotoxins including FUMs. Among them, 83 maize samples, 21 maize silage samples and three cereal grain samples were analyzed for levels of FUMs. FUMs were detected in 59% of the maize samples with 1885 μg/kg as the maximum level and in 53% of maize silage samples with 108 μg/kg as the maximum level. There were no positive cereal grain samples. The contamination concentration of FUMs in all of the positive samples was lower than the EC’s guidance values. In Germany, 84 maize samples were collected in 2006 (44 samples) and 2007 (40 samples). In all, 34% of maize samples were contaminated with FUMs in 2006, and no samples were contaminated with FUMs in 2007 [43]. Researchers estimated that the samples collected in 2006 were contaminated with a higher level of FUMs than the ones in 2007 because of the high temperature and low rainfall during anthesis and early grain filling. In 2006, FB_1_ was detected in 34% of samples with 1910 μg/kg as the mean concentration, and FB_2_ was detected in 23% of samples with 460 μg/kg as the mean concentration. Zachariasova et al. [38] reported that 11 maize silage samples were contaminated with a mean concentration of 35 μg/kg and 26 μg/kg for FB_1_ and FB_2_, respectively. In that study, FUMs were mainly found in maize, maize silage and maize-based DDGS. In our previous study, separately performed as part of another research project with different samples in 2012 [44], the results were similar to the results of this study; most compound cattle feed was contaminated with FUMs (98%). Compound feed for early broilers was contaminated with a level of mean contamination of 3459 μg/kg and a range of 333–12,823 μg/kg. The range of contamination levels was between 0.059 and 8.239 for feed ingredient samples (83.3%).

In the present study, feed ingredients were collected in 2011, 2012, 2014 and 2016 in Korea. The incidence of FB_1_ was 14.3, 70.4, 63.6, 33.3 and 37.5 in grains, grain by-products, meal, fibrous feed and food by-products, respectively. FB_2_ contaminated 48.1, 45.5, 44.4 and 12.5% of grain by-products, meal, fibrous feed and food by-products, respectively. FB_1_ and FB_2_ were found in 56.4 and 40.0% of feed ingredient samples. Kosichi et al. [39] reported that 59% of 83 maize samples were contaminated with a mean of 1885 μg/kg FUMs. In this study, the incidence of FUMs was 92.3% in 26 samples consisting of corn gluten feed, corn bran, corn gluten meal and corn germ meal with mean levels of 1440.5 and 517.6 μg/kg for FB_1_ and FB_2_, respectively. The maximum detected levels of FB_1_ and FB_2_ were 6357.5 and 1798.9 μg/kg in corn bran and 4816.0 and 1662.7 μg/kg in corn gluten meal. As a result, all FUMs concentrations in feed ingredient samples were lower than the guidance limits established by the EC and Korea.

## 4. Materials and Methods

### 4.1. Chemicals and Reagents

Analytical standards of FB_1_ and FB_2_ were purchased from Sigma Chemical Co. (St. Louis, MO, USA). Acetonitrile and methanol, purchased from Honeywell Burdick & Jackson (Morris Plains, NJ, USA), were used for the extraction of FB_1_ and FB_2_. Phosphate-buffered saline (PBS; Sigma-Aldrich, St. Louis, MO, USA) was used as a buffer solution for elution from the immune-affinity column. Ortho-phthaldialdehyde (OPA; Pickering Laboratories, Mountain View, CA, USA) was used for derivatization of FUMs for fluorescence analysis. Fumoniprep (R-Biopharm^®^, Darmstadt, Germany) and FumoniTest (Vicam Co., Milford, MA, USA) were applied for solid-phase extraction and purification. A standard solution of FB_1_ and FB_2_ was diluted with 50% acetonitrile solution (acetonitrile:water = 50:50, *v*:*v*).

### 4.2. Sampling of Compound Feed and Feed Ingredients

A total of 535 animal feed samples (425 compound feed and 110 feed ingredients) produced domestically between 2011 and 2016 in Korea were collected in order to evaluate the contamination level of FUMs. These samples were provided by the National Agriculture Products Quality Management Service Experiment Research Institute. These feed samples were prepared by the general guidelines on sampling [45]. One kilogram of sample was randomly collected from every ton of compound feed and feed ingredient. Four different samples collected from the same lot were mixed and divided into another four groups according to the guidelines mentioned above. All of the divided samples of 200 g were kept at 4 °C for analysis. Table 7 and Table 8 describe detailed data related to collecting compound feed samples and feed ingredients. The classification of compound feed and feed ingredients is shown in the supplementary data (Table S1–S5).

### 4.3. Extraction and Purification

The guidelines of the International Conference on Harmonisation (ICH) were applied to measure the level of FB_1_ and FB_2_ in feed samples using HPLC [46]. The extraction solvent (acetonitrile:methanol:water = 25:25:50, *v*:*v*:*v*; 100 mL) was added to ground feed samples (20 g), and the mixture was homogenized at 7614× *g* for 10 min. The extract was filtered through filter paper (Whatman No.4, GE Healthcare Life Science, Maidstone, Kent, U.K.), and PBS solution (40 mL) was added and mixed with 10 mL of filtered extract solution. This mixed solution (10–50 mL) was loaded onto an IAC column, and the IAC column was washed with 10 mL of PBS solution before being eluted with 4 mL of solution (methanol:water = 80:20, *v*:*v*) under gravity. The flow rate was 2–3 mL/min at the purification step using an IAC for adequate reaction. This eluted solution was dried under nitrogen gas at 60 °C and re-dissolved with 0.2–0.5 mL of solution (acetonitrile:water = 50:50, *v*:*v*). The re-dissolved residues were filtered through a membrane filter (0.22-μm pore size).

The level of FUMs in animal feed was measured with HPLC analysis using an Agilent 1100 series (Santa Clara, CA, USA) including a degasser, automatic sampler, thermostated column compartment and fluorescence detector (FLD) with a ZORBAX Eclipse XDB-C18 column (4.6 mm × 250 mm, 5 μm) (Agilent, Santa Clara, CA, USA). The run time was 30 min at 30 °C. A mixture of methanol and 0.1 M monosodium phosphate (pH 3.3 adjusted with phosphoric acid) (77:23, *v*:*v*) was used as the mobile phase. A sample (20–22 μL) was injected into the HPLC system after reaction of the sample (10 μL) and OPA solution (10–12 μL) for derivatization, which was set to occur automatically by the injector. FUMs were detected at 335 nm (excitation) and 440 nm (emission). FB_1_ and FB_2_ were detected at 3.7 min and 8.7 min without a guard column and at 10.5 min and 26.7 min with a guard column, respectively.

### 4.4. Identification of FUMs by Mass Spectrometry

The determination of FB_1_ and FB_2_ was conducted using a positive electrospray ionization (ESI) acquisition technique with liquid chromatography-tandem mass spectrometry (API 4000, Applied Biosystems MDS SCIX, Foster, CA, USA). In order to separate FB_1_ and FB_2_ in LC-MS/MS analysis, a YMC-Pack pro C18 RS column (I.D. 3.0 mm × length 150 mm, 3-μm particle size, YMC, Kyoto, Japan) and gradient elution conditions were applied in this study. The flow rate was 0.3 mL/min, and the mobile phase was composed of water (HPLC grade) containing 2 mM ammonium acetate (0.1% formic acid) and acetonitrile containing 2 mM ammonium acetate (0.1% formic acid). Analysis was conducted with the following parameters: 20 psi curtain gas, medium collision gas, 4500 ion-spray voltage and 500 °C turbo gas. A sample (10 μL) was injected into the HPLC and LC-MS/MS system, and its retention time was 8.8 min for FB_1_ and 9.5 min for FB_2_. Detection and quantification for FB_1_ and FB_2_ were performed in the multiple reaction monitoring (MRM) mode. The precursor ions (*m*/*z* 722.373 and 706.353) for FB_1_ and FB_2_ were selected from the extracted ion chromatogram (XIC) of the FB_1_ and FB_2_ standard at 250 ppb. Each selected precursor ion was compared with that of FB_1_ and FB_2_ in the sample. Two product ions of *m*/*z* 334.300 and 318.000 were further examined in order to confirm FB_1_ in the standard and the sample in the MS/MS system. In addition, two product ions of *m*/*z* 336.300 and 318.300 were further checked to confirm FB_2_ in the standard and the sample in the MS/MS system.

### 4.5. Method Validation

The HPLC analytical method for FUMs was validated with parameters of linearity, LOD, LOQ, accuracy and precision according to ICH guidelines [46]. The linearity was checked with a term expressed as a regression coefficient (r^2^) after analyzing a range of FUM standard concentrations (50, 100, 250, 500 and 1000 ng/mL). The LOD was estimated at the lowest amount of FUMs that could be only detected, but not quantified, and the concentration had a signal-to-noise ratio of 3. The LOQ was calculated as the lowest amount of FUMs that could be quantitated, which was determined as the concentration of a signal-to-noise ratio of 10. Accuracy was estimated using a recovery test that analyzed blank samples spiked with a known concentration of FUMs (100, 200 and 400 μg/kg), repeatedly, and the results were expressed as a recovery ratio. A FUMs-free pig feed was used for the recovery test as a blank sample because pig feeds are composed of major components for tested feeds in an average ratio. It contains mainly corn (45%), followed by soybean meal (20%) and others (wheat, barley, wheat shorts, corn gluten meal, distiller’s dried grains, palm oil meal, calcium phosphate and syrup, etc.). The degree of repeatability was defined as precision in this study and is expressed as percent relative standard deviation (%RSD).

## Figures and Tables

**Table 1:** Guidance values for fumonisins (FUMs) in animal feed in the EU and South Korea.

Mycotoxins	Products Intended for Animal Feed	Guidance Value in mg/kg (ppm)	
		EU [27]	Korea [29]
Fumonisin B_1_ + B_2_	Feed materials		
	-maize and maize products	60	-
	-vegetable, mixed feed ingredient, food-waste feed ingredient	-	60
	Complementary and complete feeding stuffs for:		
	-pigs, horses (*Equidae*), rabbits and pet animals	5	5
	-fish	10	10
	-poultry, calves (<4 months), lambs and kids	20	-
	-poultry, ruminants (<3 months), total mixed ration for ruminants (<3 months)	-	20
	-adult ruminants (>4 months) and mink	50	-
	-complementary and complete feed stuffs for ruminants	-	50
	-other complementary and complete feed stuffs (except for premix)	-	30

**Table 2:** Summary of the method validation study.

Analytes	*R*^2^	LOD (μg/kg)	LOQ (μg/kg)	Recovery			
				Spiked Concentrations (μg/kg)	Mean Recovery (%)	SD	RSD (%)
Fumonisin B_1_	0.999	0.20	0.30	100	108.8	5.1	4.7
				200	100.0	5.0	5.0
				400	94.0	8.6	9.1
Fumonisin B_2_	0.999	0.25	0.35	100	86.4	8.5	9.9
				200	96.4	4.9	5.1
				400	99.5	12.0	12.1

*R*^2^: coefficient of determination, LOD: limit of detection, LOQ: limit of quantitation, SD: standard deviation, RSD: relative standard deviation.

**Table 3:** FUMs concentrations in various compound feed types.

Livestock	Feed Type	FB_1_			FB_2_		
		N ^(a)^	LC (%) ^(b)^	Mean (μg/kg)	N ^(a)^	LC (%) ^(b)^	Mean (μg/kg)
Beef cattle	Early beef cattle	15	0	1258.34	15	33.3	344.51
	Middle beef cattle	9	11.1	1727.82	9	11.1	195.25
	Late beef cattle	20	0	2217.01	20	15.0	360.64
	Gestating beef cattle	19	5.3	2651.61	19	15.8	310.70
	Lactating beef cattle	2	0	585.75	2	0	160.00
Dairy cows	Dairy cow in early lactation	16	0	832.20	16	56.3	74.00
	Dairy cow in mid lactation	1	0	663.35	1	0	46.68
	Dairy cow on dry	3	0	1988.56	3	0	232.60
	High yielding dairy cow	8	0	1381.31	8	12.5	212.33
	Gestating dairy cow	2	0	834.44	2	0	135.59
Calves	Early beef calf	13	7.7	1597.98	13	7.7	226.76
	Middle beef calf	17	0	3456.55	17	11.8	413.83
	Early dairy calf	1	0	36.65	1	0	14.85
	Middle dairy calf	8	0	709.93	8	50.0	127.84
	Late dairy calf	6	0	830.48	6	33.3	115.93
	Middle breeding calf	1	0	138.00	1	0	27.45
	Late breeding calf	4	0	911.03	4	0	128.33
Pigs	Early growing pig	19	15.8	1856.77	19	68.4	20.79
	Late growing pig	11	0	487.358	11	45.5	33.23
Sows	Gestating sow	22	9.1	1039.58	22	40.9	351.95
	Lactating sow	27	18.5	1394.75	27	51.9	76.22
	Breeding gilt	3	0	2032.29	3	66.7	108.13
Piglets	Sucking piglet	14	21.4	1176.00	14	71.4	15.57
	Weanling piglet	44	25	914.01	44	52.3	35.64
Poultry	Early layer chick	3	0	655.33	3	0	177.45
	Middle layer chick	16	0	948.22	16	56.3	143.32
	Late layer chick	14	14.3	1238.41	14	42.9	195.98
	Early laying hens	26	3.8	1661.30	26	23.1	150.36
	Middle laying hens	14	0	708.91	14	21.4	108.76
	Late laying hens	2	50.0	50.00	2	50.0	13.73
	Early broiler	29	6.9	1950.36	29	31.0	571.17
	Late broiler	25	12.0	1591.34	25	40.0	415.73
	Finishing broiler	2	0	346.75	2	0	135.7
	Breeding broiler	9	44.4	979.12	9	55.6	226.83

^(a)^ N: number of samples, ^(b)^ LC: percentage of left censored results. This value in this experiment is the percentage of data below the LOD [31].

**Table 4:** Concentration mean and differences of mycotoxins in compound feed.

Mycotoxins	Livestock	Conc. of Mycotoxin (μg/kg)		F	*p*
		Mean	SD		
Fumonisin B_1_	Cattle	1779.0	2612.14	2.192	0.113
	Swine	1171.1	2684.37		
	Poultry	1382.5	2141.15		
Fumonisin B_2_	Cattle	251.8	354.70	3.243	0.040
	Swine	90.5	468.04		
	Poultry	287.9	1049.79		

**Table 5:** FUMs concentrations in feed ingredients.

Class	Feed Type	Fumonisin B_1_			Fumonisin B_2_		
		N ^(a)^	LC (%) ^(b)^	Mean (μg/kg)	N ^(a)^	LC (%) ^(b)^	Mean (μg/kg)
Grain	Grain	1	100	0	1	100.0	0
	Grain products	6	83.3	14.6	6	100.0	0
Grain by-products (bran)	Corn gluten feed	2	0	1736.8	2	0	813.4
	Soybean hull	1	0	158.3	1	0	21.5
	Wheat shorts	2	50.0	168.6	2	50.0	174.3
	Cotton seeds hull	2	0	287.6	2	50.0	67.3
	Wheat bran	5	60.0	108.0	5	80.0	22.3
	Corn bran	4	0	4201.9	4	0	1073.4
	Other grain by-products	11	36.4	1016.1	11	72.7	165.6
Meal (Vegetable protein)	Soybean meal	9	66.7	47.9	9	100.0	0
	Wheat gluten	1	0	2485.7	1	100.0	0
	Corn gluten meal	11	9.1	1241.4	11	9.1	594.9
	Corn germ meal	9	0	390.9	9	11.1	110.4
	Distillers dried grains	10	10.0	1137.9	10	40.0	126.7
	Coffee meal	1	100.0	15.5	1	100.0	0
	Palm oil meal	3	66.7	20.5	3	100.0	0
	Other meal	11	81.8	36.2	11	90.9	46.3
Fibrous feed	Fibrous feed	9	66.7	354.0	9	55.6	94.7
Food by-products	Food by-products	8	62.5	50.5	8	87.5	9.4
Beans	Beans	1	0	51.0	1	0	28.0
Seeds/nuts	Seeds/nuts	2	100.0	0	2	100.0	0
Mixed formulation	Mixed formulation	1	100.0	0	1	100.0	0

^(a)^ N: number of samples, ^(b)^ LC: percentage of left censored results (<LOD).

**Table 6:** Concentration mean and differences of FUMs in feed ingredients across the years.

Mycotoxins	Year	Conc. of Mycotoxins (μg/kg)		F	*p*
		Mean	SD		
Fumonisin B_1_	2011	680.6	1200.31	2.001	0.118
	2012	947.6	1444.54		
	2014	559.2	1299.11		
	2016	155.1	458.85		
Fumonisin B_2_	2011	161.0	339.22	1.645	0.183
	2012	266.2	466.66		
	2014	177.4	324.34		
	2016	51.7	147.40		

**Table 7:** Compound feed samples between 2011 and 2016.

Livestock	Feed Type	No. of Samples				
		Total	2011	2012	2014	2016
Beef cattle	Early beef cattle	15	6	5	2	2
	Middle beef cattle	9	4	-	2	3
	Late beef cattle	20	11	6	1	2
	Gestating beef cattle	19	10	5	2	2
	Lactating beef cattle	2	-	-	-	2
Dairy cows	Dairy cow in early lactation	16	8	6	1	1
	Dairy cow in mid lactation	1	-	-	1	-
	Dairy cow on dry	3	-	-	2	1
	High yielding dairy cow	8	-	5	2	1
	Gestating dairy cow	2	-	-	1	1
Calves	Early beef calf	13	4	5	1	3
	Middle beef calf	17	7	6	1	3
	Early dairy calf	1	-	-	-	1
	Middle dairy calf	8	-	6	1	1
	Late dairy calf	6	-	5	1	-
	Middle breeding calf	1	-	-	-	1
	Late breeding calf	4	-	-	2	2
Pigs	Early growing pig	19	6	10	3	-
	Late growing pig	11	2	5	4	-
Sows	Gestating sow	22	6	10	3	3
	Lactating sow	27	12	10	3	2
	Breeding gilt	3	2	-	1	-
Piglets	Sucking piglet	14	6	5	2	1
	Weanling piglet	44	16	10	4	14
Poultry	Early layer chick	3	-	-	1	2
	Middle layer chick	16	5	6	3	2
	Late layer chick	14	6	5	3	-
	Early laying hens	26	10	10	3	3
	Middle laying hens	14	3	5	3	3
	Late laying hens	2	-	-	-	2
	Early broiler	29	11	10	4	4
	Late broiler	25	11	9	3	2
	Finishing broiler	2	-	-	-	2
	Breeding broiler	9	4	5	-	-
Total		425	150	149	60	66

**Table 8:** Feed ingredient samples between 2011 and 2016.

Class	Feed Type	No. of Samples				
		Total	2011	2012	2014	2016
Grain	Grain	1	-	-	-	1
	Grain products	6	3	2	-	1
Grain by-products (Bran)	Corn gluten feed	2	-	2	-	-
	Soybean hull	1	-	-	1	-
	Wheat shorts	2	-	1	1	-
	Cotton seeds hull	2	-	1	1	-
	Wheat bran	5	2	2	1	-
	Corn bran	4	-	2	1	1
	Other grain by-products	11	9	-	1	1
Meal (Vegetable proteins)	Soybean meal	9	3	2	3	1
	Wheat gluten	1	1	-	-	-
	Corn gluten meal	11	4	4	3	-
	Corn germ meal	9	5	2	1	1
	Distillers dried grains	10	3	6	-	1
	Coffee meal	1	-	1	-	-
	Palm oil meal	3	-	3	-	-
	Other meal	11	7	-	1	3
Fibrous feed	Fibrous feed	9	1	-	2	6
Food by-products	Food by-products	8	2	2	1	3
Beans	Beans	1	-	-	-	1
Seeds/nuts	Seeds/nuts	2	-	-	-	2
Mixed formulation	Mixed formulation	1	-	-	-	1
Total		110	40	30	17	23

## Supplementary Materials

The following are available online at www.mdpi.com/2072-6651/9/9/286/s1, Table S1: Classification of compound feeds for cattle, Table S2: Classification of compound feeds for swine, Table S3: Classification of compound feeds for poultry, Table S4: Classification of compound feeds for dairy cows, Table S5: Classification of feed ingredients.
